# The Innate and Adaptive Immune Cells in Alzheimer's and Parkinson's Diseases

**DOI:** 10.1155/2022/1315248

**Published:** 2022-09-28

**Authors:** Boyuan Huang, Yan Zhenxin, Sisi Chen, Zhenhua Tan, Zhitao Zong, Hongbo Zhang, Xiaoxing Xiong

**Affiliations:** ^1^Department of Neurosurgery, Capital Medical University Electric Power Teaching Hospital/State Grid Beijing Electric Power Hospital, Beijing 100073, China; ^2^Central Laboratory, Renmin Hospital of Wuhan University, Wuhan, Hubei 430060, China; ^3^Department of Neurology, Zhujiang Hospital, Southern Medical University, Guangzhou 510282, China; ^4^Department of Neurosurgery, Jiujiang Hospital of Traditional Chinese Medicine, Jiujiang, Jiangxi 332005, China; ^5^Department of Neurosurgery, The Second Affiliated Hospital of Nanchang University, Nanchang 330006, China

## Abstract

Alzheimer's disease (AD) and Parkinson's disease (PD) are the most common neurodegenerative disorders of the central nervous system (CNS). Increasing evidence supports the view that dysfunction of innate immune cells initiated by accumulated and misfolded proteins plays essential roles in the pathogenesis and progression of these diseases. The TLR family was found to be involved in the regulation of microglial function in the pathogenesis and progression of AD or PD, making it as double-edged sword in these diseases. Altered function of peripheral innate immune cells was found in AD and PD and thus contributed to the development and progression of AD and PD. Alteration of different subsets of T cells was found in the peripheral blood and CNS in AD and PD. The CNS-infiltrating T cells can exert both neuroprotective and neurotoxic effects in the pathogenesis and progression. Here, we review recent evidences for the roles of innate and adaptive immune cells in the pathogenesis and progression of AD and PD.

## 1. Introduction

Alzheimer's disease (AD) and Parkinson's disease (PD) are the most common age-related neurodegenerative diseases in the world [1, 2]. AD is clinically characterized by progressive cognitive impairment, irreversible loss of memory, language disorders, and impairment of visuospatial skills. The hallmarks of AD pathology include extracellular aggregation of *β*-amyloid (A*β*) plaques and intracellular formation of neurofibrillary tangles (NFTs, also called tau aggregates), which inevitably lead to death of neurons and loss of synaptic transmission in hippocampal and cortical regions responsible for memory and learning [3–6]. Unfortunately, most cases of AD develop over tens of years without obvious symptoms of clinical dementia. Clinically apparent AD begins with mild cognitive impairment (MCI), which, after an average of 10 years, gradually progresses to moderate and severe AD. On the other hand, PD is characterized by progressive degeneration and loss of dopaminergic (DA) neurons in the substantia nigra and loss of nerve terminals in the striatum, which results in rigidity, bradykinesia, resting tremor, and postural instability [7–10]. The loss of DA neurons in PD is coupled with intracellular filamentous deposits called Lewy bodies, which consist of aggregates of parkin, *α*-synuclein (*α*-Syn), phosphorylated neurofilament, and components of the ubiquitin-proteasome pathway [11, 12]. However, there are conflicting views about whether or not the large aggregates (amyloid plaques, NFTs, Lewy bodies) are responsible for neuronal death in AD and PD [12–15]. Although distinct from each other in clinical manifestations and pathological mechanisms, PD and AD are considered “protein misfolding” diseases due to deposition of improperly folded, modified protein aggregates in specific anatomical areas. Furthermore, the neurodegenerative processes in these two diseases are generally accompanied by neuroinflammation [13–16], which may play complicated roles in these diseases: both beneficial and detrimental, both cause and effect.

Neuroinflammation is a physiological response to protect CNS from exogenous and endogenous insults, but uncontrolled and prolonged inflammatory responses are detrimental to the CNS [16]. Traditionally, resident immune cells of the CNS (i.e., microglia) were thought to play a major role in this neuroinflammation [17]. Recently, it has also been demonstrated that both innate and adaptive immune cells from the periphery can penetrate through the blood-brain barrier (BBB) into the CNS and thus involved in the neuroinflammation [18, 19]. In the acute phase of neuroinflammation, these cells can exert protection by surveillance functions and maintaining homeostasis. Conversely, during chronic phase such as AD or PD, the cells may exert detrimental effect on neurons. However, how the innate and adaptive immune systems influence the onset and development of AD and PD remains elusive. Here, we review recent literatures related to the role of innate and adaptive immune cells in PD and AD.

## 2. Microglia in AD and PD

Innate immune cells include neutrophils, dendritic cells (DCs), monocytes/macrophages, natural killer (NK) cells, and other cells that are capable of rapidly generating inflammatory responses directed towards extra- and intracellular pathogens. So far as CNS is concerned, it should be extended some immune cells circulating in the cerebral spinal fluid and found in the brain (e.g., resident T cells, Astrocytes), as well as microglia, which were investigated most in CNS.

Microglia are resident immune cells which have been demonstrated to play essential roles in CNS tissue maintenance, injury response, and pathogen defense as well as recruitment of peripheral immune cells [20]. The conventional classification of microglia defined M1 phenotype as pro-inflammatory and neurotoxic, and M2 phenotype as anti-inflammatory and neuroprotective [21, 22]. Recently, accumulating evidence challenged this simplified classification and suggested microglia showed a range of intermediate states expressing mixed phenotypes in neurodegenerative diseases.

**Figure 1:**
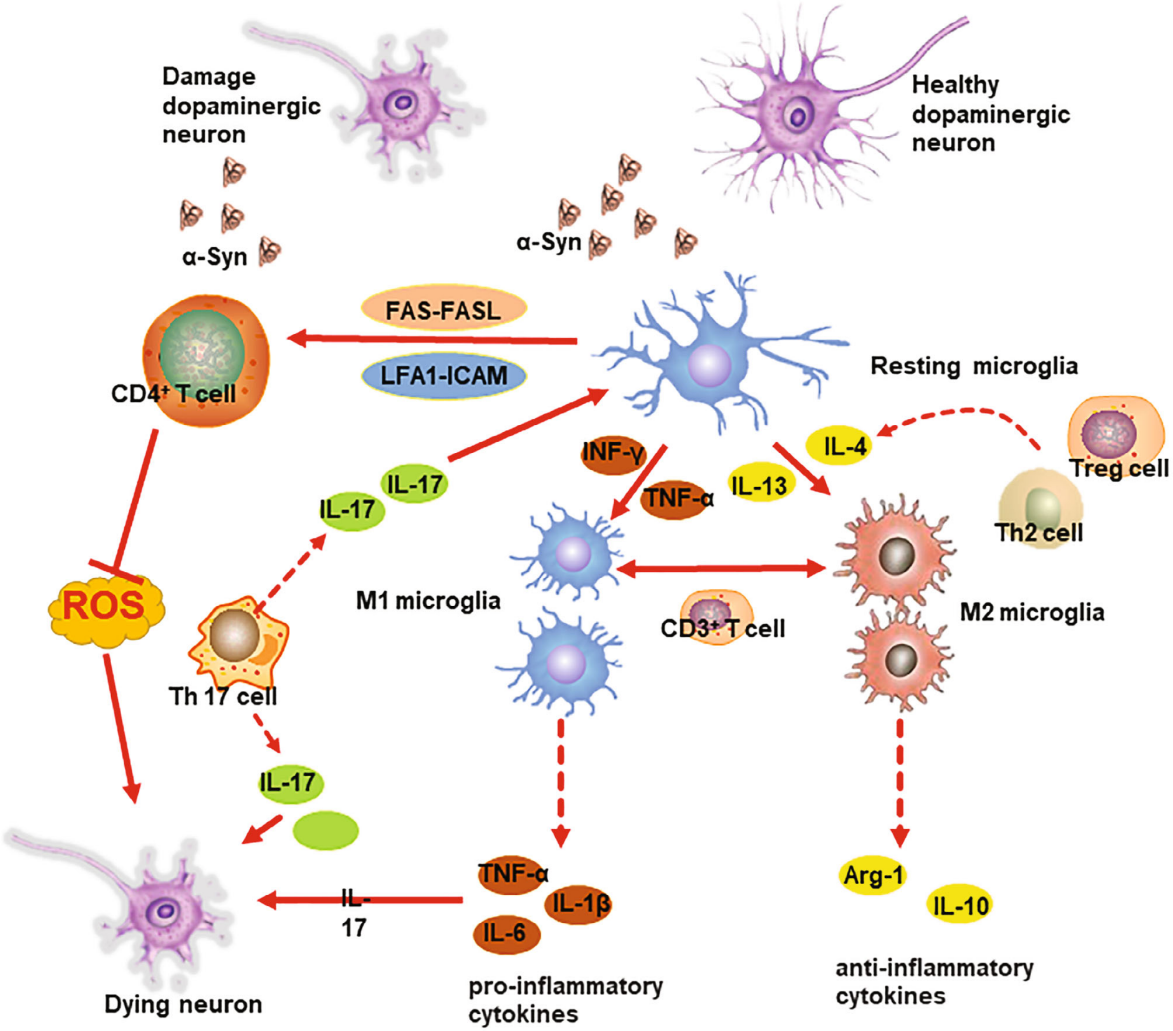
Potential mechanisms of T cell contributions to PD pathogenesis. T cells may directly contribute to PD pathogenesis through interaction of LFA1 with ICAM or by Fas-FasL signaling. T cells can also indirectly influence PD pathogenesis by mediating microglial transformation from a M2 phenotype (anti-inflammatory) to M1 phenotype (pro-inflammatory). Moreover, *α*-Syn deposits may be presented to activate T cells, thus initiating an autoimmune inflammatory response, which in turn exacerbates PD pathology by disturbing the balance of effector Th subsets. The production of IL-4 and IL-13, inducing alternative activation of microglia—known as the M2 state—can exert a protective effect against neuronal damage.

Recent evidence suggested that activated microglia can form a protective barrier around amyloid deposits making it less toxic, which can not only prevent new A*β* accumulation but also reduce axonal dystrophy in the nearby neuropil [23]. It was demonstrated that in the early stage of AD, activated microglia can serve as neuroprotective by releasing several proteases for clearance of A*β* plaque [24]. Moreover, the M2 phenotype was found to help in phagocytosis of A*β* plaques and creates a physical barrier to prevent further spreading of plaque [23]. Actually, acute microglia activation appeared to be neuroprotective, but the sustained chronic microglial activation seemed to be detrimental. Accordingly, such unbridled microglial malfunction may lead to overproduction of pro-inflammatory cytokines which will result in a shift towards M1 polarization contributing to the neurotoxic effect and synaptic loss resulting from the A*β* accumulation [25]. In addition, activated microglia can release some neurotoxic inflammatory cytokines such as TNF-*α* and INF-*γ*. However, their downstream signaling involves activation of MAPK and NF-*κ*B which usually exerts neuroprotective effect [26]. With respect to PD, *α*-Syn was found to promote microglial phagocytic activity which was associated to the DA neurons loss in vivo [27, 28]. In PD patients, a negative correlation was observed between the microglial phagocytosis marker CD68 and the disease duration [29]. Interestingly, CD68 was overexpressed at early stage implying an increase in phagocytosis when cells start to die and tissue clearance is needed. Moreover, CD68 positive cells were found increased over the course of PD in substantia nigra (SN) which showed a more amoeboid cell shape suggesting that these cells were also in a pro-inflammatory state rather than just clearing dead cells [30]. Thus, whether a beneficial or harmful role that microglia plays in neurodegenerative diseases depends on their functional status (Figure 1).

Numerous receptors have been demonstrated to be involved in microglial stimulation and subsequent secretion of inflammatory cytokines. In the context of AD and PD, some members of the Toll-like receptor (TLR) family, which can recognize protein aggregates in these diseases, are involved in the disease progression [31]. As far as AD is concerned, TLRs have been demonstrated to contribute to the pathology by upregulation of mRNA levels in mice [32, 33] and different mechanisms activated by A*β* in microglial cells. For example, TLR2 and TLR4 expressed on microglia have been shown to play significant roles in the clearance of protein deposits in neurodegenerative disorders [32]. It has also been shown that plaque-associated microglia can upregulate mRNA expression of TLR4 and TLR2 in a mouse model of cerebral amyloidosis [34]. TLR4 has been verified to have beneficial roles in AD pathophysiology through A*β* phagocytosis in APP/PS1 mice [35]. Moreover, TLR4 stimulation can attenuate tauopathy in human tau transgenic mice [36] and TLR2 blockade can reduce gliosis and A*β* burden with associated improvement in learning in APP/PS1 mice [37]. In a recent study, TLR5 has been validated upregulated in the frontal cortex of moderate AD cases. GAS6, one ligand of TAM (Tyro3, Axl, Mer) family which play crucial roles in limiting inflammatory responses upon TLR stimulation, has also been demonstrated to exert negative impact on AD progression. In this study, the author found that upregulation of GSA6 induced by co-stimulation with A*β* and flagellin in THP-1 cells could be prevented by neutralization of TLR5 [38]. This study demonstrated presence of an immunosuppressive response in moderate AD cases, arguably mediated through the TAM system, and the potential implication of TLR5 signaling, upon prolonged immune stimulation in the presence of A*β*. In addition, this evidence also suggested that “TLRs” are engaged in disease progression inhibition.

It has been suggested that TLR4 also plays beneficial roles in PD by inducing *α*-Syn clearance from microglia, both in PD patients and mouse models [30, 39–41]. In addition, TLR2 has also been shown to be upregulated in microglia in both PD patients and transgenic Thy1.2-*α*-Syn mice [42–44]. Recently, several studies suggested that *α*-Syn secreted by neurons could stimulate TLR2 and induce subsequent inflammatory responses in microglia, thus contributing to neurodegeneration [44, 45].

As mentioned above, AD and PD are regarded as “protein misfolding” diseases due to characteristic aggregation of improperly folded modified proteins. The extracellular aggregation of A*β* is believed to be a crucial pathogenic mechanism in AD, resulting from an imbalance in the production versus clearance of A*β*, in which microglia may play an important role [46, 47]. Excessive production of amyloidogenic A*β* has been regarded as a major cause of early-onset AD (EOAD) [46, 47].

Genome-wide association studies (GWAS) have identified over 20 single-nucleotide polymorphisms (SNPs) that are strongly related to AD risk in late-onset AD (LOAD), also called sporadic AD, which constitutes the majority of cases [48, 49]. Intriguingly, most of the identified risk genes for AD are expressed preferentially or selectively in microglia compared to other types of cells in the brain [50, 51]. In addition to these common variants, it has also been demonstrated that microglia can abnormally express some rare genetic variants related to AD [52, 53]. For example, a missense mutation (R47H) in triggering receptor expressed in myeloid cells 2 (TREM2), a cell surface protein highly and selectively expressed on microglia, was found to increase the risk of AD approximately three-fold [54, 55]. Traditionally, stimulation of microglial TREM2 through interaction with the activating adaptor protein DAP12 has been thought to initiate signal transduction pathways associated with enhanced proliferation, chemotaxis, and phagocytosis [56, 57]. TREM2 binds to apolipoprotein (APOE), encoded by the APOE gene, which is known to contribute to a significant fraction of the heritable risk for late-onset AD [57]. In addition, overexpression of wild-type TREM2 was sufficient to enhance uptake of low-density lipoprotein (LDL), Clusterin (CLU) (identified by unbiased protein microarray screen) in heterologous cells, whereas TREM2 disease variants were impaired in this activity. TREM2 knockout microglia showed reduced internalization of LDL and CLU. A*β* binds to lipoproteins and this complex is efficiently taken up by microglia in a TREM2-dependent fashion [58].

Consistent with these observations, researchers have found that TREM2-deficient microglia show diminished A*β* internalization in vivo and decreased uptake of A*β*-lipoprotein complexes in vitro [55, 59]. However, the impact of TREM2 deficiency has been unclear because studies have shown both increased and reduced A*β* deposition in TREM2-deficient AD mouse models [44, 56, 60]. A recent study using the APP/PS1-21 mouse model showed that TREM2 deficiency could ameliorate A*β* aggregation early in the disease but was associated with exacerbation in the late stage of disease [61]. In addition, it has been shown that microglia could form a protective barrier around A*β* aggregates and force amyloid fibrils into a tightly packed and potentially less toxic form, thereby reducing the accumulation of new A*β* deposits onto existing plaques and ameliorating axonal injury in nearby neurons [23]. Overall, studies related to TREM2 have so far suggested several mechanisms by which microglia may protect against A*β* aggregation and development of AD: phagocytosis of insoluble fibrillar A*β* aggregates, clearance of soluble A*β* species, and sequestration and compaction of A*β* plaques.

In addition to TLRs and TREM2, genome-wide association study (GWAS) has identified cluster of differentiation 33 (CD33) as another immune receptor conferring risk for AD. CD33 shows elevated expression in AD and functions as a modifier of microglial stimulation, thereby inhibiting A*β* clearance [62]. It has also been shown that the CD33 risk allele is associated with elevated expression of TREM2 on mononuclear phagocytes [63]. There is also strong evidence that elevated expression of CD33 is related to greater disease burden in PD [64].

Recently, it has been shown that the stimulation of microglia by *α*-Syn can in turn promote *α*-Syn phagocytosis [65]. Moreover, *α*-Syn phagocytosis can also be facilitated by leucine-rich repeat kinase 2 (LRRK2), a regulator of microglial response [66]. LRRK2 is one of the most commonly mutated genes in both familial and sporadic PD and can influence microglial internalization and degradation of *α*-Syn, thus exacerbating microglial pathology mediated by *α*-Syn [66].

Another crucial signaling pathway involved in microglial stimulation in PD is the nuclear factor-kappa B (NF-*κ*B) pathway [67], which can promote microglial secretion of pro-inflammatory cytokines such as TNF-*α* and interleukin-1*β* (IL-1*β*) [68]. Cytokines released by stimulated microglia can attract peripheral immune cells to the brain.

Some studies have demonstrated that overexpression of *α*-Syn can induce microglial expression of major histocompatibility complex-II (MHC-II), which plays an essential role in both innate and adaptive immune responses in PD [69]. In addition, microglia can also express multiple receptors for neurotransmitters, including glutamate, acetylcholine, gamma-aminobutyric acid, norepinephrine, and cannabinoid receptors, all of which can exert neurotoxic or neuroprotective effects [21]. For example, cannabinoid receptor CB2 was found to be overexpressed in animal models of PD, conferring a neuroprotective effect [70, 71]. Moreover, it has also been demonstrated that cannabinoid agonists can exert protective effects against nigrostriatal neuronal loss in the 1-methyl-4-phenyl-1,2,3,6-tetrahydropyridine (MPTP) mouse model of PD [72]. Another study found that senile plaques in AD patients express cannabinoid 1 (CB1) receptor together with markers of microglial activation, and that CB1-positive neurons, present in high numbers in control cases, are greatly reduced in areas of microglial activation. Cannabinoid agonists can inhibit A*β* toxicity in vivo as well as A*β*-induced microglial activation in vitro [73].

Together, both TLR family and CD33 are involved in the regulation of microglial function during the pathogenesis and progression of AD and PD. TREM2 and LRRK2, respectively, impact the microglial function in AD and PD. Moreover, microglia can also express multiple receptors for neurotransmitters to exert neurotoxic or neuroprotective effects. All these findings suggest that microglia play a role as double-edged sword in both the pathogenesis and progression of AD and PD. Targeting the regulation of microglial functions may be a promising therapeutic strategy.

## 3. Peripheral Innate Immune Cells in AD and PD

AD and PD have been historically viewed as diseases restricted to the brain even though neuroinflammation was regarded as a crucial component of these neurodegenerative disorders. Recently, the observation that the integrity of the BBB is compromised in neurodegenerative diseases provided a clue to explain the migration of peripheral immune cells into the brain [74]. Importantly, these immune cells may not only infiltrate into the CNS but may also return to the peripheral circulation, thus establishing a cycle between the periphery and the brain. It has already been established that a continuous cross-talk between the brain and the peripheral immune system exists [75]. This relationship suggested a further hypothesis that neurodegenerative disorders such as AD and PD are systemic diseases in which the immune system exerts an influence on pathogenesis and progression.

### 3.1. NK Cells

As described above, the innate immune cell population includes NK cells, neutrophils, dendritic cells (DCs), monocytes/macrophages, and other immune cells. NK cells are potent cytotoxic effectors against pathogen-infected cells and tumor cells [75–77]. NK cells play a crucial role in bridging the innate and adaptive immune systems by secreting multiple cytokines and interacting with other immune cells. It was shown that NK cell populations did not differ among AD patients and age-matched healthy elderly controls [77]. However, there is evidence of increased NK cell activity in patients with amnestic mild cognitive impairment (aMCI), an antecedent to AD [76]. For example, elevated levels of TNF*α*, Interferon*γ* (IFN*γ*), and granzyme B coming from NK cells were observed in aMCI, compared with those of confirmed mild AD (mAD) patients. Cluster of differentiation 95 (CD95), a prototypical death receptor belonging to the TNF receptor superfamily that regulates tissue homeostasis through induction of apoptosis, was also observed to be increased in mAD and amnestic mild cognitive impairment (aMCI). Chemokine ligand 19- (CCL19-) dependent chemotaxis was reported to be decreased in aMCI and mAD patients, while Chemokine C Receptor 7 (CCR7) expression was increased in aMCI [76, 77]. We suggest that this state of activation reflects an innate immune response against an undefined challenge and may contribute to neuroinflammation. According to this assumption, rather than serving a protective role, NK cells may contribute to pro-inflammatory conditions in the setting of AD.

In other words, NK cells in peripheral blood of PD patients were found increased compared to those of non-PD patients [78]. Moreover, NK cells were also found in the PD mouse brain and substantia nigra of postmortem PD patients [79]. Recently, NK cells were demonstrated to efficiently internalize and degrade *α*-Syn aggregates via the endosomal/lysosomal pathway. Human NK cells were found capable to scavenge various forms of *α*-Syn species. Within NK cells, *α*-Syn aggregates are degraded and cytoplasmic *α*-Syn is colocalized with endosomal and lysosomal protein markers, implicating NK cells in the clearance of extracellular *α*-Syn [80]. It was also worth noting that NK cell cytotoxicity was attenuated and IFN-*γ* secretion was decreased along with the presence of *α*-Syn aggregates [80]. In addition, NK cells can interact with microglia to trigger NK cell-mediated cytotoxicity towards hyperactive microglia which is induced by sustained *α*-Syn burden [81]. Recently, an analysis about peripheral immune cells in PD observed an elevation in less cytotoxic NK cells and a decline in highly cytotoxic NK cells along with disease progression. Accordingly, this implies that NK cells may initially play a protective role in PD by the clearance of *α*-Syn pathology, which may further be lost with disease progression and decreasing NK cell activity [82].

### 3.2. Monocytes/Macrophages

**Figure 2:**
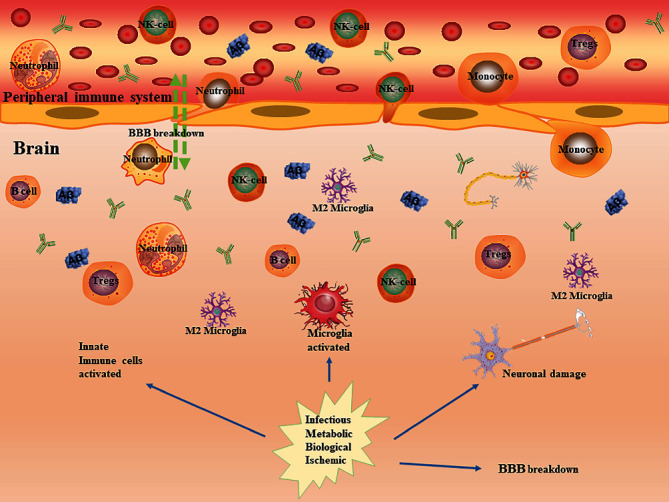
Peripheral innate immune cells and cerebral innate immune cells involved in the pathogenesis of AD. Insults begin in the brain along with impaired BBB function, promoting release of inflammatory mediators to the periphery. Peripheral innate immune cells become stimulated and infiltrate into the brain. These cells can either help to resolve or perpetuate inflammation. They can return to the periphery where they may amplify the inflammatory process.

In the blood, three subsets of monocytes have been identified based on the surface markers CD14 and CD16: the classical monocytes (CD14^++^/CD16^−^), the intermediate monocytes (CD14^++^/CD16^+^), and the non-classical monocytes (CD14^+^/CD16^++^) [83]. In neurodegenerative diseases such as AD and PD, the CNS is damaged which usually leads to an increase in the BBB permeability and thus favoring the peripheral monocytes infiltration. It has been demonstrated that A*β* can induce chemokine release such as monocyte chemoattractant proteins (MCPs) which can attract monocytes. After that, monocytes start to produce pro-inflammatory cytokines like IL-6 and TNF-a and improve their phagocytic capacity of toxic elements, including A*β* [84]. A recent study showed that A*β*-stimulated cell cultures from AD patients contained elevated numbers of inflammatory monocytes/macrophages. These cells could express TLR2, TLR4, IL-6, and CCR2, which could in turn promote the migration of monocytes/macrophages through the blood-brain barrier (BBB) into the brain [82, 83] (Figure 2).

The three subsets of monocytes may play different roles in AD. Non-classical monocytes (CD14^+^/CD16^++^) are observed decreased in AD patients compared to aMCI patients or healthy people suggesting a protective role of this subset in this disease [85]. Similarly, a progressive reduction of classical monocytes was observed in AD patients, also suggesting a more prominent role in this disease [86]. Moreover, a dysregulation of the monocyte subset distribution was also found in this study. However, it remains to be clarified whether this dysregulating distribution resulted from a shift of the monocyte phenotype or a progressive death of classical monocytes.

With respect to PD, monocytes were supposed to be a contributing role to DA neuronal loss [87]. Several studies have demonstrated an enrichment of classical monocytes in peripheral blood of PD patients, especially in those with a high risk of developing early dementia (HR-PD), of which monocytes express higher levels of TREM2 [88, 89]. Moreover, classical monocytes that express CCR2 were found to be enriched in peripheral blood of PD patients along with a strong reduction of CCR2-positive cells [90]. In addition, it was observed a relationship between CCR2+ monocytes and disease duration, suggesting a crucial role of the activation of CCL2-CCR2 axis in PD [91]. Considering CCR2 is important for peripheral monocytes to recruit to inflamed tissue, that the deletion of CCR2 failed in protecting DA neuronal loss in MPTP mouse model may suggest a potential neurotoxic role of monocytes [92]. Another remarkable phenomenon was that P11 protein, which is involved in depression, was found to be expressed almost 10-fold higher in monocytes than in the other leukocytes in PD patients who was experiencing depression many years after disease diagnosis. However, in the PD patients without depression, the expression of P11 protein in monocytes was not observed at the same high levels [93]. Accordingly, the P11 protein could be a potential biomarker for assessing the severity of PD, especially in those patients with depression.

### 3.3. Polymorphonuclear Neutrophils

The roles of polymorphonuclear neutrophils (PMNs) in the pathogenesis of AD have also been recently investigated. Neutrophils may exert effects during the initiation phases of AD pathogenesis, although their role is controversial. Some studies reported reduced production of superoxide anion and diminished phagocytic capacity in AD, while others showed an increase [94]. Another study observed altered PMN function between healthy elderly individuals, aMCI subjects, and mAD patients [85]. For instance, CD177 was found to be overexpressed in mAD patients, while CD14 and CD16 expressions were reduced in PMN of mAD [77]. These data suggest altered PMN function in aMCI and mAD, which may be attributable to the variety of pathological stimuli associated with AD development.

With respect to PD, the number of neutrophils was found elevated in the peripheral blood of PD patients which seemed to occur years before diagnosis [95]. Several studies also found increased neutrophils in the peripheral blood during prodromal and clinical PD which is correlated with different symptomatic presentations [96, 97]. However, it remains to be clarified the role of the neutrophils in PD.

In summary, resident microglia have multiple roles in the CNS, including protecting the brain from various insults such as infection and injury and regulating inflammatory responses. Peripheral innate immune cells including NK cells and monocytes/macrophages have been demonstrated to interact with the brain. Considering the view that microglia are responsible for the maintenance of homeostasis and protection against pathogens, it is reasonable to hypothesize that sustained neuroinflammation plays a role in the pathogenesis of neurodegenerative diseases. This sustained neuroinflammation may result from the failure of microglia to either recruit innate immune cells to promote and sustain their activities or to terminate their activation after pathogens are eliminated.

## 4. Adaptive Immune Cells in AD and PD

Adaptive immunity includes humoral immunity dependent on the specific recognition of antigens by B cells and cellular immunity dependent on specific recognition of T cell receptors. Based on specific types of activities, T cells can be further categorized into CD8+ cytotoxic T (Tc) cells, which function to eliminate infected somatic cells, and CD4+ T helper (Th) cells that help regulate other immune cells. Moreover, Th cells can be further subdivided into different effector T helper (Th) subgroups: immunosuppressive regulatory T cells (Tregs), pro-inflammatory Th9 cells, pro-inflammatory Th17 (secreting IL-17) and Th1 (secreting IFN*γ*) cells, and anti-inflammatory Th2 cells (secreting IL-4).

Increasing evidences suggest that T cells play crucial roles in the pathogenesis and progression of AD and PD. Studies of lymphocyte populations in blood from AD patients showed an elevated frequency of CD4+ T helper cells and reduced frequency of CD8+ cytotoxic T cells [98, 99]. However, a reduced frequency of CD4+ T helper cells and elevated frequency of CD8+ were detected in blood from patients with PD [98]. Another study reported that Tregs expressing fork head box P3+ (FoxP3+) and CD4+ were also increased in AD patients [100]. Moreover, Th9 cells and Th17 cells have also been found to be increased in AD patients [101]. Hypothesizing that Th17 cells and Tregs represent pro- and anti-inflammatory populations, respectively, these findings demonstrate that adaptive immune cell populations are altered in AD patients.

Analysis of peripheral blood from patients with PD showed distinct alterations in frequencies of lymphocyte subpopulations, which also suggested stimulation of adaptive immunity in PD pathogenesis. Some researchers studied the phenotype of circulating lymphocytes in 30 untreated and 34 levodopa-treated patients. They found that Th cells were decreased and activated, and CD4+CD25+ lymphocytes were increased which were independent of levodopa treatment [102]. Other researchers have found that CD4+ Th cells were decreased in PD patients' peripheral blood, of which CD4+CD29+ cells and CD4+CD45RO+ memory T cells were more decreased. Moreover, the reduction of CD4+CD29+ cells was correlated with clinical stage of PD [103]. Another study reported a lower ratio of IL-4/IFN*γ*-secreting Th cells in 33 PD patients, implying a shift towards a Th1-type immune response [104]. Moreover, elevated frequencies of IL-17-secreting cells were found in 29 PD patients and 18 patients with early PD, indicating a potential role for Th17 cells in the development and progression of PD [78, 105]. Tregs have also been demonstrated to be involved in the progression of PD. Some authors defined CD4+CD25+ cells as Tregs and found that these cells were decreased in PD patients [102, 104]. However, when FoxP3 and CD127 expression were taken into account, the frequency of Tregs defined as CD4+FoxP3+ or CD4+CD25+CD127- showed no differences between PD patients and healthy controls [106, 107]. Intriguingly, although not reduced in quantity, Tregs from PD patients in the aforementioned study showed impaired capacity to suppress proliferation of effector T cells [107]. Numerous factors may influence findings related to investigation of blood cell populations in PD patients, including patient age, medication use, and genetic variants [108–113].

Alterations of T cells in the peripheral blood of PD patients suggested a significant role in PD pathology, which gave rise to investigations on T cell infiltration into the CNS in human postmortem brain tissues and animal models of PD. Using the MPTP model of PD, Sommer et al. recently demonstrated that Th cells could cause neuronal death in the SN through factor-associated suicide/factor-associated suicide ligand (Fas/FasL) signaling [114]. Moreover, in the Thy1-WTS transgenic mouse model, CNS-infiltrating CD3+ T cells were demonstrated to contribute to PD pathogenesis by mediating microglial transformation from the anti-inflammatory M2 phenotype to the pro-inflammatory M1 phenotype, which were able to reduce microglial clearance of *α*-Syn deposits and thus promote *α*-Syn pathology [115]. Furthermore, this research team found that Th17 cells isolated from PD patients could aggravate neuronal cell death in co-cultures of iPSC-derived midbrain DA neurons [116]. Using the MPTP mouse model, it was recently demonstrated that Th17 cells could directly contact neurons through adhesion molecules and thus induce neurotoxicity [117]. So far as CD8+ T cells were concerned, the role remains unclear. It was found that CD8+ T cells infiltrated into the brain in response to increased *α*-Syn levels, which was early in the disease course prior to significant DA loss [118, 119]. This suggested that CD8+ T cells may contribute little to neuronal cell loss, but may contribute neuroinflammation in response to pathogenic changes of *α*-Syn aggregation.

Apart from neurotoxicity, T cells may also exert neuroprotective effects in neurodegenerative disease. Tregs have been demonstrated in vitro to suppress release of reactive oxygen species (ROS) from microglia and thus prevent ROS-induced neuronal damage [120]. This could explain, to some extent, how adoptively transferred activated Tregs could confer protection against neurotoxicity in another study using the MPTP mouse model [121]. Furthermore, a recent phase I clinical trial in a small cohort of PD patients has started to investigate sargramostim, a human recombinant granulocyte-macrophage colony stimulating factor that can stimulate Tregs-mediated suppression [122]. Excitingly, this trial has shown that sargramostim treatment for PD patients could boost the frequency of Tregs among total CD4+ T cells and enhance their suppressive capacity. More importantly, patients showed improvement on the unified PD rating scale III and magnetoencephalography-recorded cortical motor activities [122]. From another perspective, these observations also support the hypothesis that PD pathogenesis may result at least in part from an imbalance between anti- and pro-inflammatory effector T cell subsets.

The effects of CNS-infiltrating T cells in AD appear to be complex. It has been demonstrated that T cells may be neuroprotective through release of neurotrophic factors, stimulation of microglial phagocytic activity, and assistance in reducing A*β* deposition [123]. However, some A*β*-reactive T cells may also exacerbate AD progression through secretion of pro-inflammatory cytokines, thus leading to chronic inflammation [123]. Moreover, in the APP/PS1 mouse model of AD, Th1-released IFN*γ* was shown to impair cognitive function by promoting microglial stimulation and increasing A*β* aggregation. Treatment with an anti-IFN*γ* antibody could alleviate disease progression in APP/PS1 mice, which supported the view that Th1 cells may exert a neurotoxic effect in AD pathology [124]. In addition, A*β*-specific Th2 cells can prevent the production of cytokines by glial cells, and A*β*-specific T1 cells possess the properties of inducing the production of pro-inflammatory cytokines by microglial cells [123]. According to these evidences, we speculate that different stages of AD progression have distinct profiles of T cell subpopulations and that the immune cells may play contradictory roles at different stages of AD.

These evidences suggest that alteration of T cells in the peripheral blood in AD and PD plays a significant role in pathology. And CNS-infiltrating T cells in AD and PD can exert both neuroprotective and neurotoxic effects.

## 5. Conclusion

Taken together, numerous sources of evidences demonstrated the contributions of innate and adaptive immune cells to the pathology of neurodegeneration in AD and PD. Numerous evidences suggested that TLR family, TREM2, CD33, and LRRK2 were involved in the regulation of microglial function in the pathogenesis and progression of AD or PD, making it as double-edged sword in these diseases. Altered function of peripheral innate immune cells has also been demonstrated in AD and PD which can interact with the brain, and modulating the functions of these cells may become a beneficial approach to modify the progression of neurodegeneration. Alteration of T cells in the peripheral blood has been found in AD and PD, and CNS-infiltrating T cells can exert both neuroprotective and neurotoxic effect in the pathogenesis and progression. In addition, an imbalance of Tregs and pro-inflammatory T (Th17 or Th1) cells, which can impair neuroprotective effects and induce neuronal damage, is associated with neurodegeneration. Thus, developing compounds that target peripheral innate immune cells or promote expansion of neuroprotective Tregs and anti-inflammatory T cells may also be promising approaches for the treatment of AD and PD, as examples of neurodegenerative diseases with underlying neuroinflammation.
